# Predictions of response to temperature are contingent on model choice and data quality

**DOI:** 10.1002/ece3.3576

**Published:** 2017-11-15

**Authors:** Etienne Low‐Décarie, Tobias G. Boatman, Noah Bennett, Will Passfield, Antonio Gavalás‐Olea, Philipp Siegel, Richard J. Geider

**Affiliations:** ^1^ School of Biological Sciences University of Essex Colchester UK; ^2^ Instituto de Investigaciones Marinas (IIM‐CSIC) Vigo Spain

**Keywords:** biogeography, biotrait, global change, niche, thermodynamic, warming

## Abstract

The equations used to account for the temperature dependence of biological processes, including growth and metabolic rates, are the foundations of our predictions of how global biogeochemistry and biogeography change in response to global climate change. We review and test the use of 12 equations used to model the temperature dependence of biological processes across the full range of their temperature response, including supra‐ and suboptimal temperatures. We focus on fitting these equations to thermal response curves for phytoplankton growth but also tested the equations on a variety of traits across a wide diversity of organisms. We found that many of the surveyed equations have comparable abilities to fit data and equally high requirements for data quality (number of test temperatures and range of response captured) but lead to different estimates of cardinal temperatures and of the biological rates at these temperatures. When these rate estimates are used for biogeographic predictions, differences between the estimates of even the best‐fitting models can exceed the global biological change predicted for a decade of global warming. As a result, studies of the biological response to global changes in temperature must make careful consideration of model selection and of the quality of the data used for parametrizing these models.

## 1 INTRODUCTION

Temperature is one of the most important environmental drivers of physiology and thus has important implications for the biogeography of all organisms and how they will respond to global environmental change. Predicting the biological response to changes in temperature is thus a key endeavor in biology, and thousands of studies have measured the response of biological processes to temperature. Data on the temperature response of over 200 traits covering a wide taxonomic breadth (>300 species across all domains of life) have been compiled (Dell, Pawar, & Savage, 2011; Gillooly, 2001; Parent & Tardieu, 2012). Even for a single trait and a single group of organisms, for example phytoplankton growth rate, over 200 studies have been inventoried (Thomas, Kremer, Klausmeier, & Litchman, 2012; Thomas, Kremer, & Litchman, 2016). These datasets have been used to establish fundamental metabolic scaling rules (Dell et al., 2011; Gillooly, 2001) and biogeographic theories (Seto & Fragkias, 2007). In addition, temperature response curves, whether derived from in situ measurements of abundance along natural temperature gradients or from in vitro measurements from laboratory experiments, are used extensively for the prediction of the effects of climate change on the biogeography of organisms [e.g., (Beaugrand, Goberville, Luczak, & Kirby, 2014)], the risks of extinctions (e.g., Sinervo et al., 2010), and global biogeochemical cycling [e.g., (Cox, Betts, Jones, Spall, & Totterdell, 2000)]. These essential predictions depend on our ability to accurately and precisely model temperature response and parameterize these equations for a large variety of traits and a diversity of species.

Currently, there is no consensus on the “best” equation to employ for modeling the thermal response of abundance and/or metabolic rates, and it is likely that different processes require different equations. Here, we review the equations available for modeling the thermal response and test them on highly resolved measurements for seven phytoplankton species and published data covering a diversity of physiological traits across a large taxonomic breadth. We used subsampling from the highly resolved phytoplankton growth measurements to assess the effect of data quality on the error in the estimate of temperature response parameters and rates. The results of this analysis were used to establish nominal data quality requirements and to include robustness in the choice of equations. The effect of model choice and data quality is then compared to the amount of change predicted in the biogeography of a phytoplankton in response to global warming.

### 1.1 Review of temperature response equations

The features of the temperature response that is of paramount importance include the cardinal temperatures that define the temperature range (*T*_min_, *T*_max_), the optimum temperature at which the response is maximal (*T*_opt_), and the sensitivity of the response to temperature change around *T*_opt_ or as the temperature of the environment approaches *T*_min_ or *T*_max_. In addition to three equations of response to suboptimal temperatures (*T*_min_ to *T*_opt_, Equations 1–3, Supporting Information), at least 12 different equations have been proposed to account for the temperature dependence of growth rate, metabolic rates, or abundance across the full range from *T*_min_ to *T*_max_ (Table 1, Equations 4–15). Different equations may lead to different predicted responses to global warming or imply that different mechanisms underlie the temperature response. Furthermore, different traits (e.g., growth and speed of movement) have different activation rates, curvature, and skew (Dell et al., 2011), although these differences depend both on model choice and on data quality (Pawar, Dell, Savage, & Knies, 2016). It has also been suggested that activation rates differ between taxa, but that these differences are also partly dependent on the equation used (Chen & Laws, 2016).

**Table 1:** Nonexhaustive list of equations that have been employed to describe the relationship between growth or metabolic rates and temperature across the full response range

Formula	Equations	Number of parameters	References
$\text{Rate}=a\cdot exp\left(\frac{-b}{R\cdot T}\right)-c\cdot exp\left(\frac{-d}{R\cdot T}\right)$	4	4	(Li & Dickie, 1987) citing (Hinshelwood, 1947)
$\text{Rate}=\frac{a\cdot T\cdot exp\left(\frac{-b}{R\cdot T}\right)}{1+exp\left(\frac{-c}{R}\right)\cdot exp\left(\frac{-d}{R\cdot T}\right)}$	5	4	(Li & Dickie, 1987) citing (Johnson, Eyring, & Williams, 1942)
$\text{Rate}=\;\frac{a\cdot \left(\frac{T}{298.15}\right)\cdot exp\left(\frac{b}{R}\cdot \left(\frac{1}{298.15}-\frac{1}{T}\right)\right)}{1+exp[\frac{c}{R}\cdot \left(\frac{1}{d}-\frac{1}{T}\right)]+exp\left[\frac{e}{R}\left(\frac{1}{f}-\frac{1}{T}\right)\right]}$	6	6	(Heitzer et al., 1991)
$\text{Rate}=\;\frac{a\cdot \left(\frac{T}{293.15}\right)\cdot exp\left(\frac{b}{R}\cdot \left(\frac{1}{293.15}-\frac{1}{T}\right)\right)}{1+exp\left[\frac{c}{R}\cdot \left(\frac{1}{d}-\frac{1}{T}\right)\right]}$	7	4	(Montagnes et al., 2008) citing (Schoolfield, Sharpe, & Magnuson, 1981)
$\text{Rate}=a\cdot exp\left[-0.5\cdot {\left(\frac{\left[T-T_{\mathrm{ref}}\right]}{b}\right)}^{2}\right]$	8	3	(Li & Dickie, 1987) citing (Stoermer & Ladewski, 1976)
$\text{Rate}=a\cdot exp\left[-0.5\cdot {\left(\frac{abs\lceil T-T_{\mathrm{ref}}\rceil }{b}\right)}^{c}\right]$	9	4	(Montagnes et al., 2008)
$\text{Rate}=a\cdot \exp\left(c\cdot T\right)\left[1-{\left(\frac{T-T_{\mathrm{ref}}}{b}\right)}^{2}\right]$	10	4	(Thomas et al., 2012) citing (Norberg, 2004)
$\text{Rate}=a+b\cdot T+c\cdot T^{2}$	11	3	(Montagnes et al., 2008)
$\text{Rate}=\;\frac{1}{1+\left(a+b\cdot T+c\cdot T^{2}\right)}$	12	3	(Montagnes et al., 2008) citing (Flinn, 1991)
$\text{Rate}={\left[a\cdot \left(T-T_{\min}\right)\right]}^{2}\cdot {\left[1-exp\left(b\cdot \left(T-T_{\max}\right)\right)\right]}^{2}$	13	4	(Ratkowsky et al., 1983)
$\text{Rate}=a\cdot \left\{1-exp\left[-b\cdot \left(T-T_{\min}\right)\right]\right\}\cdot \left\{1-exp\left[-c\cdot \left(T_{\max}-T\right)\right]\right\}$	14	5	(Kamykowski, 1986)
$\text{Rate}=\;R_{\max}\cdot {\left\{sin\left[\pi \cdot {\left(\frac{T-T_{\min}}{T_{\max}-T_{\min}}\right)}^{a}\right]\right\}}^{b}$	15	5	(Boatman et al., 2017)

*R* = Universal gas (Boltzmann) constant.

A number of studies have tested the quality of a few of these equations for a specific process (e.g., growth rate or photosynthesis) and species (Angilletta, 2006; Li & Dickie, 1987; Montagnes, Morgan, Bissinger, Atkinson, & Weisse, 2008). In these studies, model selection was based on a measure of equation fit to the data (e.g., likelihood) with a penalty for the number of parameters (e.g., by use of the Akaike information criterion –AIC‐). In addition to likelihood‐based selection, one needs to consider the accuracy of the estimates of key parameters such as the cardinal temperatures (e.g., the optimum, minimum, and maximum temperatures *T*_opt_, *T*_min_, *T*_max_) and the robustness of these estimates to changes in data quality. For example, equations with few parameters that assume a symmetric response around *T*_opt_ would underestimate the *T*_opt_ of a negatively skewed response but may still have the lowest AIC (be selected as the “best” equation) for datasets with few measurements.

**Figure 1:**
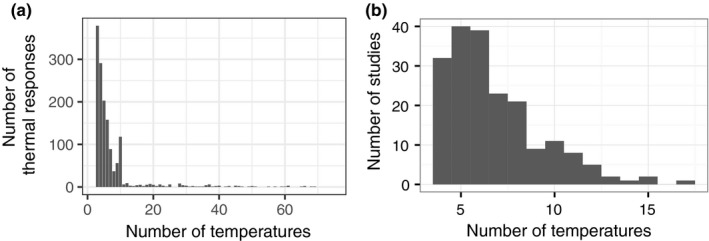
Characteristics of existing datasets for the determination of thermal response curves. (a) Number of temperatures in the most comprehensive meta‐analysis database currently compiled, excluding studies with two or fewer temperatures and three studies with more than 75 temperatures (Dell et al., 2013). Median and mean number of temperatures is 3 and 5.7, respectively. 71% of temperature responses only cover the supra‐ or suboptimal part of the temperature range and 84% do not have more than 7 temperatures and thus cannot be used to parameterize all equations in Table 1. (b) Number of temperatures in each study of the growth response of phytoplankton to temperature (Thomas et al., 2012). The median number of temperatures is 6 and 69% of temperature responses do not have more than 7 temperatures and cannot be used to parameterize all equations in Table 1. A large proportion of studies do not cover supra‐ and suboptimal temperature ranges

Both the temperature range and/or the temperature resolution of experimental or observational studies may be constrained by logistical considerations and/or experimental goals (Figure 1). These constraints on data quantity and quality can affect model selection and the associated mechanistic biological interpretations of fitted parameters such as the activation energy, which provides an index of the increase in performance with increasing temperature when temperature is suboptimal (Knies & Kingsolver, 2010; Pawar et al., 2016).

Even the minimal requirement to avoid overfitting, that the number of temperatures measured must exceed the number of parameters in an equation, is often not met. There is a risk that fundamental postulates, such as the existence of a strong relationship between microbial biogeography and thermal niche, and predictions of the response to global change may be biased by fitting equations to data of insufficient quality. This is because estimates of the numerical values of equation parameters are expected to depend on both the temperature resolution of the data and the location (relative to *T*_opt_) and extent of the temperature range (relative to *T*_min_ and *T*_max_) over which data are collected. However, the effect of data quality on the inferences that can be made when modeling temperature response across the range from *T*_min_ to *T*_max_ has not been tested previously.

Although suboptimal temperature responses are usually explained by thermodynamic activation and have been extensively studied, several putative mechanisms are proposed for the supra‐optimal decline in biological activity and these remain to be extensively tested. The decline can be attributed to the denaturation of one or more rate limiting enzymes (Corkrey, Olley, Ratkowsky, McMeekin, & Ross, 2012). However, enzyme denaturation usually occurs at much higher temperatures than the optimal temperature for most physiological rates measured. The decline in rate at supra‐optimal temperatures for individual enzymes (Hobbs et al., 2013) or bulk processes (Schipper, Hobbs, Rutledge, & Arcus, 2014) may be explained by changes in heat capacity of the system driven by protein dynamics (the number of available modes associated with covalent bonds). Ecological explanations have also been suggested for the supra‐optimal decline, as temperature alters abiotic and biotic conditions. For example, gas solubility decreases with temperature. Increasing temperature could thus lead to increasing CO_2_ limitation for photosynthetic processes in aquatic photoautotrophs or increasing oxygen limitation for respiration across all aquatic organisms (Pörtner, 2010; Pörtner & Knust, 2007). This limitation could potentially extend to terrestrial organisms in terms of changes in partial pressure with temperature, but findings are inconclusive (Klok, Sinclair, & Chown, 2004).

Several equations have been proposed to model the full functional response of biological rates to temperature from the minimum to maximum temperatures that will support growth (Table 1, nonexhaustive and new models emerging, DeLong et al., 2017). Small differences in the shape of the response curve can have major implications for predicting performance in the field [reviewed in (Dowd, King, & Denny, 2015)] and for interpretation of the mechanism(s) driving the activation and deactivation process. Four of the 12 equations in Table 1 are based on thermodynamics of chemical reactions (Equations 4, 5, 6, 7, review of equations for enzyme‐catalyzed reaction rates in (DeLong et al., 2017)] and involve various combinations of exponential dependencies on temperature. Two other equations that include exponential functions make no claim to a mechanistic underpinning and are purely empirical (Equations 11, 12). Equations 8 and 9 are modifications of a Gaussian function, while Equations 13 and 14 are second‐order polynomial, and all four are again strictly empirical. Finally, the last equation in Table 1 (Equation 15) is also empirical but uses the sine function. Some of the simpler equations (three parameters) are symmetric around the optimal temperature, but most equations presented can capture the commonly observed negative skew found in temperature response curves (steeper inactivation at temperatures above *T*_opt_ than activation at temperature below *T*_opt_).

The first attempts to quantify the functional response of rate (**μ**) to temperature (T), the **μ**‐T curve, were based on analogies between microbial growth rates and chemical reaction kinetics. Recent studies suggest that all biological growth rates can be modeled as if growth is controlled by the activation and denaturation of a single limiting enzyme (Corkrey et al., 2012). The simplest of these (Equation 4) assumes that the observed rate is the difference between two opposing processes, both of which follow the Arrhenius equation; in this equation, the coefficients within the exponential functions are activation energies. When applied to a chemical reaction, the parameter “*a*” is a rate constant with units of inverse time per degree Kelvin (e.g., s/°K), *b* = ΔH^‡^ (enthalpy of activation; units of kilocalories/mole), *c* = ΔH (enthalpy of reaction; units of kilocalories/mole), *d* = ΔS (entropy of reaction; units of kilocalories/mole per °K). An earlier equation (Equation 5) describes the situation where active and thermally denatured forms of an enzyme exist in a reversible thermodynamic equilibrium. The most complicated of these equations is the “master equation” (Equation 6) of Heitzer, Kohler, Reichert, and Hamer (1991), which assumes that the active form of the rate‐limiting master enzyme is in equilibrium with two inactive states that result from high‐temperature or low‐temperature denaturation. When low‐temperature denaturation is excluded, this master equation simplifies to Equation 7. In both Equations 6 and 7, “*a*” is the rate at the reference temperature of 298.15°K (=25°C).

Despite clear deviations from this pattern, including skew, modeling the temperature dependence of biological rate as a Gaussian distribution (Equation 8) has been attractive to ecologists in part because of its simple parameterization (Angert, Sheth, & Paul, 2011; Dowd et al., 2015). The Gaussian equation may be specifically suited to modeling aggregated responses that are the sum of individual responses. For example, although it may not be an adequate equation for the temperature response for a single species, it may be the correct equation for the response of a community that consists of many species with different values of *T*_opt_. Equation 8 describes a normal distribution, where the parameter “*a*” is the rate at the optimal temperature (*T*_opt_) which is found at the midpoint of the temperature range and the parameter “*b*” is the standard deviation (also in units of temperature). Montagnes et al. (2008) modified this equation to obtain a modified Gaussian function that allows for the asymmetry around the optimum temperature often seen in the **μ**‐T curve (Equation 9).

Thomas et al. (2012) referencing (Norberg, 2004) multiplied the quadratic by an exponential function to obtain Equation 10. In this equation, there is a reference temperature (*T*_ref_) that determines the location of the maximum of the quadratic portion of the function. This is a generalization of the function proposed by Norberg (2004) in which the values of “*a*” and “*c*” were based on the Eppley function (*a* = 0.59/d; *c* = 0.0633/°C).

All of the equations considered to this point were either based on theoretical considerations related to chemical reaction kinetics (Equations 4–7) or allowed direct estimation of ecologically relevant parameters such as *T*_opt_ or the thermal niche width (Equations 8–10). Two other equations do not have a theoretical basis nor do they allow ecologically relevant temperatures to be estimated directly. These are based on a second‐order polynomial (Equations 11, 12) (Montagnes et al., 2008).

None of the equations examined to this point include the lower and upper temperature limits for biological rates (*T*_min_, *T*_max_) as fitted parameters. However, *T*_min_ and *T*_max_, along with the temperature at which the biological rate is maximum (*T*_opt_) are the cardinal temperatures that are often of most interest to ecologists. Some of these equations may be reformulated to include some of the cardinal temperatures, for example Equation 10 to include *T*_min_ and *T*_max_ (Baker et al., 2016). For equations lacking specific cardinal temperatures, the cardinal temperatures can be estimated from the fitted equation (see Methods section).

Finally, we turn to three equations where *T*_min_ and *T*_max_ are among the parameters found directly in the equation (fitted parameters), rather than needing to be calculated from the equation. These are the empirical equations of Ratkowsky, Lowry, McMeekin, Stokes, and Chandler (1983) (Equation 13) and Kamykowski (1986) (Equation 14), and an empirical equation that is a modified sine function Boatman, Lawson, and Geider (2017) (Equation 15). The modified sine function also returns the maximum rate (*R*_max_) at the optimum temperature as a directly fitted parameter, and *T*_opt_ can be calculated from the other fitted parameters. This equation also includes parameters that characterize the skewness (*a*) and kurtosis (*b*).

This is not a comprehensive account of all available equations to equation temperature response. Some equations have been proposed for the purpose of simulation and are difficult to fit to data (e.g., Follows, Dutkiewicz, Grant, & Chisholm, 2007). Other equations are minor variations of equations we have included [e.g., (Beaugrand et al., 2014) contains an equation that is comparable to Equation 8].

## 2 MATERIAL AND METHODS

### 2.1 Measurement of phytoplankton growth rate

We measured the temperature dependence of growth rate for seven taxonomically distinct phytoplankton. Growth rates were measured at a high‐temperature resolution (in 0.4–0.5°C increments) with extensive thermal coverage on either side of the temperature optima (18–39 individual temperatures per species; with at least two temperatures with positive growth on either side of the optima). The different species provide different expected temperature optima, skew, and spread on which to test the equations (specific rates reported in Fig. S1).

The species assayed include a coccolithophorid, *Emiliania huxleyi* (CCMP 370); a cyanobacterium *Trichodesmium erythraeum* IMS101; and two diatoms, *Thalassiosira pseudonana* (CCMP 1335); *Phaeodactylum tricornutum* (CCMP 2561); two chlorophytes *Dunaliella tertiolecta* (CCAP1320) and *Pycnococcus provasolii (CCMP1203)*; and a prymnesiophyte, *Isochrysis galbana (Ply 546)*. Specific details of the media and light for each species are provided in the data file. The number of replicates at each temperature is in parenthesis next to each genus below.

Growth rates for *Trichodesmium* [published previously in (Boatman et al., 2017)], *Emiliania, Thalassiosira,* and *Phaeodactylum* were measured using the method described by (Boatman et al., 2017). Briefly, cultures were grown at low volumes (5 ml) in 12 ml glass test tubes in a thermal gradient block (temperature is controlled at both ends of an aluminum block using circulating water baths and a linear temperature gradient forms across the block). As a proxy for biomass, daily measurements of fluorescence (*F*_*o*_) were made on dark‐adapted cells (20 min) using a FRRfII Fastact Fluorometer (Chelsea Technologies Group Ltd, UK). Cultures were kept at the lower section of the exponential growth phase and optically thin to avoid nutrient limitation, self‐shading and to minimize CO_2_ drift.

For *Dunaliella* (*rep=2*), *Pycnococcus* (*2*) and *Isochrysis* (*2*) cultures were grown in 24‐well microtiter plates sealed with air permeable membranes. Similar to cultures that were grown in glass test tubes, these plates were also grown on a thermal gradient block (described above). The surface of the gradient was covered with 1 cm of water to enhance thermal conductance between the block and the well plates. Growth of the cultures was assessed by a daily measurement of optical density at 660 nm using a multiparameter plate reader (FLUOstar Omega).

Growth was monitored during early exponential growth phase, and the exponential growth rate (μ) was calculated from the slope of the natural log of fluorescence or the natural log of optical density as a function of time.

### 2.2 Published data

In order to provide a robust test of the thermal response between taxa and allow for a comparison of fit between traits, we supplemented our measured data (described above) with existing published data. We used the biotraits database (Dell, Pawar, & Savage, 2013), a database of temperature response in phytoplankton growth (Thomas et al., 2012), and additional data from the literature (sources cited in data file). Datasets with positive rates for at least seven different temperatures with at least two temperatures being above and two being below the optimal temperature were selected from the databases. Datasets were not selected based on our proposed data quality requirements (see section on “Data quality requirements” in the results section below) as too few datasets met these more stringent requirements.

### 2.3 Equation fitting

We implemented the fitting of all equations in an R package available on Comprehensive R Archive Network (CRAN temperatureresponse). The equations were fit to data using a modified Levenberg–Marquardt algorithm (Elzhov, Mullen, Spiess, Bolker, & Mullen, 2015; More, 1978). This algorithm allows robust fitting of nonlinear equations, even when reliable starting parameters cannot be established. When equation parameter values represent features of the dataset, the starting values were estimated from the dataset (e.g., the *a* in Equations 8–10 was set as the maximum rate in the dataset, *T*_ref_, *T*_opt_, *T*_min_, *T*_max_ were set to the mean, the median, the minimum, and the maximum temperature of the dataset, respectively). When this was not possible, starting values for the parameters were the fitted parameters from the source publications for the equation, or a parameter set that ensured a downward parabola‐like shape. In equations requiring inputs in °K, values were converted in the equation from °C. The equations were fit to positive nonzero data averaged across replicates at each temperature. This is essential for equations with either asymptotic or exponential relationships of rate with temperature at the extremes, because zero values reported from above *T*_max_ or below *T*_min_ have high leverage on the equation fit and lead to poor predictions within the biokinetic range. For appropriate equation fits, the only null rates that should be included are *T*_max_ and *T*_min_, which cannot be determined before fitting. As a result, no zero values were kept. However, measurements extending to the limits of the growth range, that is, including zero values, would be necessary for the most accurate parametrization of some equations.

From equation fits, cardinal temperatures were extracted (Sinclair et al., 2016). These included:

*T*_opt_: the temperature at which the maximum rate is predicted to be achieved, which was determined using numeric optimization.
*T*_50 min_ and *T*_50max_: the lowest and highest temperatures at which 50% of the maximum rate is predicted to be achieved. This was calculated as the roots of the function when 50% of the predicted maximum rate was removed (R package rootSolve).
*T*_min_ and *T*_max_ (CT_min_ and CT_max_): temperatures within which a positive rate is predicted. This was calculated as the roots of the function. Some equations are asymptotic and therefore would not predict zero or negative rates, in which case *T*_min_ and *T*_max_ cannot be determined.

Activation and deactivation rates were calculated from the mean of value of the derivative across sub‐ (*T*_min_ to *T*_opt_) and supra‐ (*T*_opt_ to *T*_max_) optimal temperatures, respectively. Skew was calculated as the difference between activation and deactivation (i.e., a negative skew indicates that deactivation is steeper than activation).

Equations were ranked on each dataset using Bayesian information criterion (BIC). The difference between equations in model quality across datasets was tested using a Kruskal–Wallis rank sum test on BIC‐based ranks followed by the associated post hoc pairwise comparison (Giraudoux, 2017; Siegel & Castellan, 1988). The same conclusions arise when other measures of model quality were used; values for Akaike information criterion (AIC) and the AIC corrected for finite sample sizes (AICc) are available in supplemental material (Fig. S2).

Reported deviations in cardinal temperatures were calculated as the difference from the weighted mean across all equations (weighted by Akaike weights). Reported deviations in growth were calculated absolute deviation from the weighted mean across all equations (weighted by Akaike weights).

Differences between the different equations in their prediction of cardinal temperatures were assessed using analysis of variance (ANOVA) and a Tukey‐HSD. An ANOVA and a Tukey‐HSD were also used to compare equations for the temperature range required to stay within the designated thresholds for deviation from the fit to the full data (0.5°C for *T*_opt_ and 5% for growth rate). Differences between equations for sample size required to stay within these thresholds were assessed using a generalized linear equation (GLM) with a log‐link for the Poisson distribution of count data and Tukey contrasts.

To assess similarity between equation predictions across the temperature range, the Euclidian distance was calculated based on the rate predicted by the equation at each experimental temperature and clustering was done using *Ward's* minimum variance method (Fig. S3).

### 2.4 Data quality sensitivity analysis

To ensure that the high‐resolution datasets were of sufficient quality to distinguish between equations, we conducted a simulation based on equation fits to each dataset. Normally distributed random noise was added to the predicted growth rate value from each equation at each temperature. The noise was centered on 0 and its standard deviation was the square root of the mean residuals squared arising from the fit of the equation. Each equation was then fit to the simulated datasets generated by each equation and ranked based on BIC. Each simulation was replicated five times.

To measure sensitivity of the estimate for *T*_opt_ and the estimate of growth rate at each temperature to the temperature resolution of a dataset, a decreasing proportion of the measured temperatures were removed based on: (1) random sampling across the temperature range to establish the number of temperatures required and (2) limiting the temperatures included in the analysis to those where the observed growth rates were above a predetermined proportion of the maximum growth rate, thus capturing a proportion of the temperature range. A range of 100% is expected to extend from *T*_min_ to *T*_max_, while a range of 50% includes temperatures allowing at least 50% of the maximum growth rate to be achieved (from CT_50 min_ to CT_50 min_). *T*_opt_ is expected to always be within the temperature range of the data sampled using a proportion of the maximum growth rate.

Data quality requirements for precision and accuracy of *T*_opt_ and the estimate of growth rate at each temperature were assessed by fitting the equations to subsamples of the phytoplankton growth datasets and comparing these values to values obtained from fits to the complete data. Error was measured as the absolute deviation compared to values obtained from the fits to the complete dataset of cardinal temperature measurements (*T*_opt_) and the mean deviation in predicted rate at all temperatures. The temperature response of each individual species was treated as a replicate in this analysis, and confidence intervals were calculated across these replicates. An error of 0.5°C in *T*_opt_ or an average error of 5% of the maximum growth rates was set as the minimum quality thresholds. The critical number of temperatures was defined as the maximal number of temperatures at which the threshold was exceeded plus 1. The critical range was the maximum range at which the threshold was exceeded or met. In some cases, this was the lowest value for number of temperatures or range at which equations could be fit to the subsampled data.

### 2.5 Predicting changes in biogeography with global warming

Given the centrality of these equations (Table 1) to the prediction of the biotic response to global warming, tour aim was to assess whether differences among the equations used to account for the temperature dependence of growth rate can affect predictions of the effect of global warming on the biogeography of phytoplankton. To do this, we make the simplifying assumption that the geographical range of a species depends on the response of its growth rate to temperature. Sea surface temperature (SST) data were used to model the distribution of a species based on the response of its growth to temperature. Each equation was parameterized using the experimental data for the species, and the parameterized equation was applied to prediction of growth from SST.

Contemporary SST for the month of August for the years 2006 to 2016 was obtained from MODIS data accessed using the Giovanni online data system, developed and maintained by the NASA GES DISC (Acker & Leptoukh, 2007). Predicted SST for August 2100 was obtained from NCDC‐NOMADS. This predicted SST was based on IPCC SRESA1B emission scenario for CO_2_ emissions and modeled using the Geophysical Fluid Dynamics Laboratory (GFDL) Coupled Climate Model (CCM 2.1) (Delworth et al., 2006). Values from the month of August are used as an example, and similar observations would be made if another month of the year was selected or if calculations were based on mean annual temperature, although the latter would not account for seasonality.

We recognize that any inferences based on such an analysis are subject to the caveats that (1) phytoplankton abundance may not correlate with growth rate, (2) biogeography is affected by many other factors that may change in concert with or independent of global warming, and (3) given their rapid growth rates, phytoplankton can be expected to evolve in response to sustained warming.

## 3 RESULTS

### 3.1 Differences between equations

**Figure 2:**
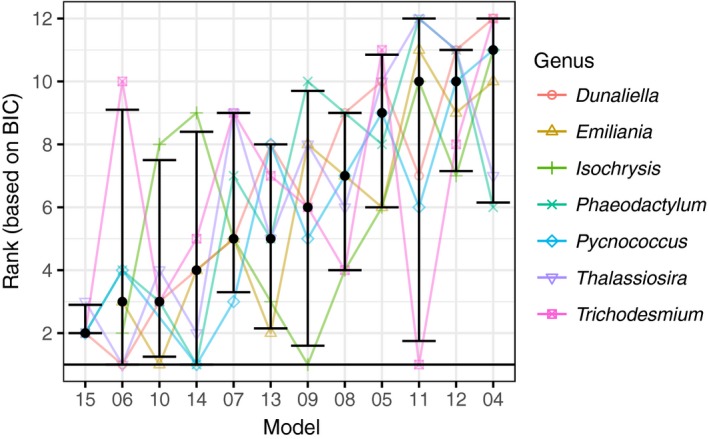
Equation ranking based on BIC for each dataset. Equations are ordered by median rank (best equations at left with lower rank). Point is the median rank and error bars are 95% confidence interval across datasets

All equations could be fit to each phytoplankton growth dataset, but no single equation consistently provided the best fit (i.e., could not account for the majority of variance) across all phytoplankton growth datasets (Figure 2). Most equations could not be distinguished across datasets based on rank, although Equations 6, 14, and 15 had better ranks than 4, and Equation 15 also significantly outranked 12 (*p* < .05, Figure 2).

Simulations indicate that the quality of the phytoplankton growth datasets is sufficient to for the selection of a best model. All equations had better rankings on the simulated data that they had generated than on data generated by any other equation (Kruskal–Wallis *p* < 10^−3^, Fig. S4).

**Figure 3:**
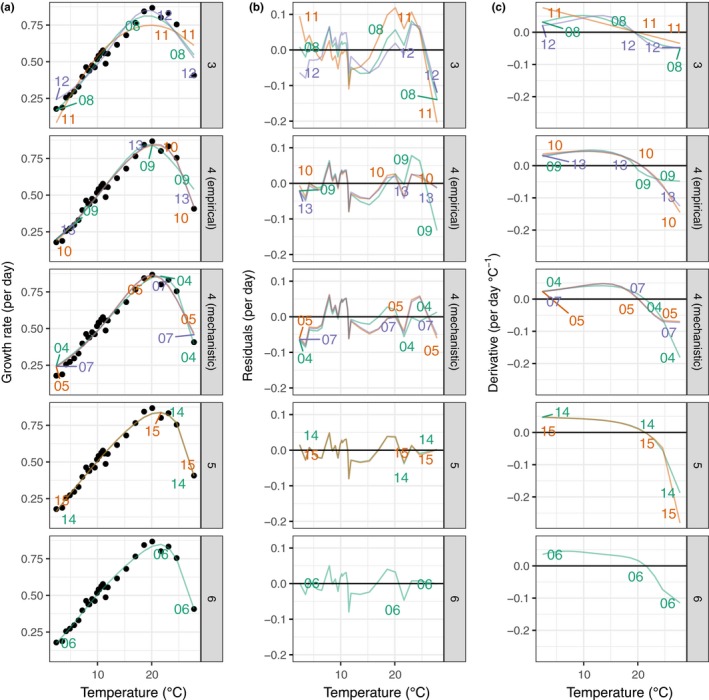
(a) Equation fit to an example dataset of phytoplankton growth rate as a function of temperature (Phaeodactylum tricornutum). The points are the measured growth rate (same values across panels), and the lines are the equation predicted growth rates. (b) Equation residuals as function of temperature. (c) Value of the first derivative (gradient) at each measured temperature. Numbers within the figure indicate the equation number. Equations are grouped as a function of their number of parameters (3–6). Equations with four parameters are further divided between empirical and mechanistic equations to minimize clutter within the plots. Lines for individual equations are labeled with color and the equation number. Similar patterns can be observed for other species

For a given dataset, the 12 equations (Table 1) did not converge on the same optimal temperature or maximum growth rate (Figure 3). Predicted optimal temperatures were on average −1.18°C [range from −2.28 to −0.18°C] from the weighted mean (Akaike weights) predicted optimal temperature across equations (S4), and the mean absolute deviation in growth rate at each temperature was 0.018 day^−1^ [range 0.015 day^−1^ to 0.022 day^−1^] when compared to the weighted mean across equations. Equations 4 and 6 consistently predicted higher optimal temperatures compared to other equations. Equations with a high number of parameters (5–6) led to similar predictions, but equations based on similar mechanisms, similar functional forms, or similar rank in terms of BIC did not lead to more similar predictions (Fig. S3).

Equations differed in their skew (deviation from median skew across equations, *F*_11,81 _= 2.87, *p* < 0.01), with the average skew being −0.017 [−0.030, −0.005] across all equations and datasets. As a consequence, *T*_50 min_ and *T*_50max_ were highly variable between equations and datasets. The median distance between equations for each dataset was 1.0°C for CT_50min_ and 2.9°C for CT_50max_. However, for some of the species in our dataset, some equations (Equations 6, 7, 12, and 14) produced estimates greater than 10°C from the weighted mean value across equations for these cardinal temperatures.

**Figure 4:**
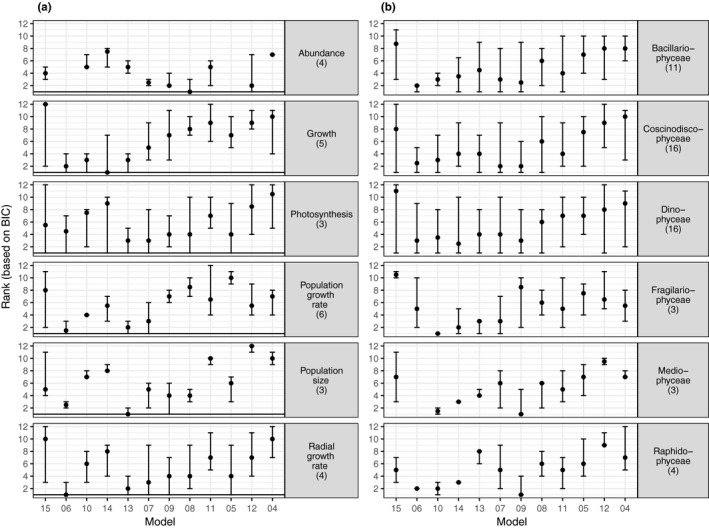
Equation rank based on BIC across (a) trait type [data compiled in (Dell et al., 2013)] and for (b) growth rate across algal classes or phyla [data compilation of (Thomas et al., 2012)] for each equation. Only traits or classes/phyla with more than two taxonomic units are included in the figure. Points indicate the median and the error bars indicate the 95% confidence interval calculated across experiments (a single taxonomic unit can be in multiple experiments). Equation order is based on median equation rank for the phytoplankton growth dataset (as in Figure 2). Numbers in parentheses indicate the number of taxonomic units (up to species when identified) within each trait or class. Not all equations converged on a solution for all individual published datasets

There was no individual equation that outperformed all other equations consistently across or within traits, nor within an algal class (for growth rate) where there was taxonomic replication (Figure 4). All equations represent the best equation for at least one of the responses (for a trait of a given taxa), except for Equation 4 which performed poorly in general.

### 3.2 Data quality requirements

**Figure 5:**
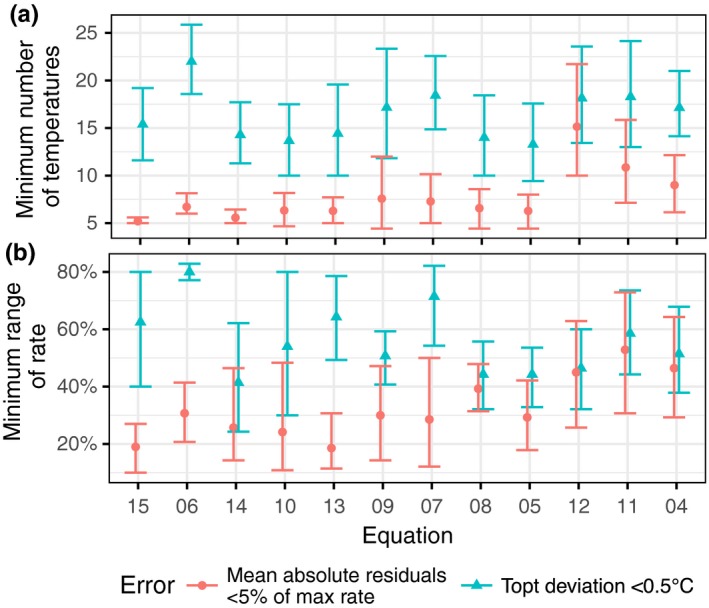
(a) Number of temperature points and (b) the range in growth rate required to maintain the predicted *T*_opt_ within 0.5°C of the value predicted on the full dataset (blue triangles), and to maintain rate predictions on average within 5% of the value predicted from the full dataset (red circles) for each equation. Equations are ordered based on median rank in the full datasets (matching Figure 2)

For all equations (Table 1), there was an approximately linear increase in the error of cardinal temperatures estimates with a decrease in temperature resolution (i.e., number of experimental temperatures). Similarly, the error increased linearly with a decrease in the measured range of growth rates (difference between the minimum rate in the subsample and maximum rate). Only the most extreme equations differed significantly in terms of their data quality requirements for number of temperatures. On average across all equations, a minimum of 16 [range of 15–17] temperature points are required to maintain the predicted *T*_opt_ within 0.5°C of the value predicted on the full dataset including all temperatures measured (Equation 6 differed from 5, 8, 10, and 14, *p* < .05, Figure 5a). A minimum of 8 [7–9] temperature points was required to maintain predictions of growth rate to within 5% of the value predicted from the full dataset (Equation 4 differed from Equation 8 *p* < .05). For the range in rates measured, 56% [50%–60%] of the full range (0 to maximum rate) is required to maintain the predicted *T*_opt_ within 0.5°C of the value predicted on the full dataset and 29% [24%–34%] maintain predictions of growth rate to within 5% of the value predicted from the full dataset (Figure 5b). Based on BIC, some of the “best” fitting equations require data of the highest resolution and range in order to maintain the quality of their fit (e.g., Equation 6 had the highest number of temperatures for the accurate prediction of *T*_opt_), while some of the weakest fitting equations are the most robust to loss in data quality (e.g., Equation 5), although these differences are only marginal.

### 3.3 Implications for predictions of the response to global warming

**Figure 6:**
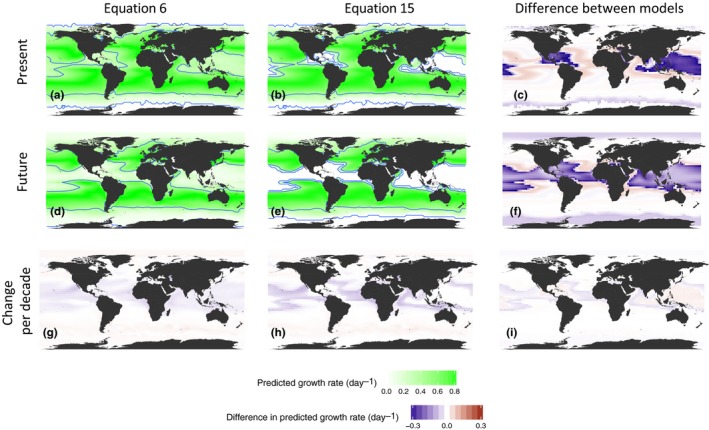
Biogeographic distribution Phaeodactylum tricornutum based on two best‐fitting equations (Equation 6 a,d,g and Equation 15 b,e,h) applied to sea surface temperature for August, in present day (average from 2006 to 2016, a–b) and modeled for the future (averages for 2095–2105, d–e); the average change in growth per decade (additive, not compounded) for the period 2006–2016 to 2095–2105 (g–h); and the difference between the predictions from these two equations for growth (c–f) and for change (i). Blue contour lines for growth are for CT_min/max_ and T_50min/max_. This is not intended to be an accurate representation of the biogeography of P. tricornutum. Rather, it is provided to illustrate the scale of differences between equations, and we note that similar differences between equations arise independent of species

We found that differences among equations in the predicted growth rates for our studied phytoplankton species translate into large differences in expected contemporary biogeography even when the two best fitting equations are compared. Equations 6 and 15 have similar quality scores (Figure 2) and lead to similar predications of rate (Fig. S3); however, Equation 6 predicts a global mean growth of 1.9% less than Equation 15 (Figure 6a–c). This difference is comparable to the change predicted from a decade of global warming. Based on a projection of future SST, all equations lead to predictions of large‐scale changes in biogeography for the studied species; however, the magnitude of change differs between equations. For example, the global mean decline in growth for *P. tricornutum* of 20.75% over the modeled period or 2.3% per decade with Equation 6 and 25.5% over the period or 2.9% per decade for Equation 15, Figure 6g–h).

## 4 DISCUSSION

### 4.1 Scale of difference in predicted biogeography and response to global warming

The difference between equations for predicted rates (mean difference of 0.018 day^−1^ across the temperature range for growth of our phytoplankton) and cardinal temperature (mean difference in *T*_opt_ of 0.44°C for growth of our phytoplankton) may be perceived as small but are ecologically significant. When scaled to predictions of changes in global processes, such as biogeography, differences between the best models can be larger than changes predicted over decades (Figure 6). The importance of data quality and modeling approach is recognized across disciplines which attempt to predict responses to global change. Differences in datasets and methodology can lead to opposing predictions of the change in biogeography with global warming (Brown et al., 2016). Changes in the scale of the observed difference between equations can alter predictions of species extinction or changes in the epidemiology of major diseases (Mordecai et al., 2013).

The difference between equations in global average predicted rates and our threshold for data quality are both on the order of responses to global climate change, including observed changes in terrestrial primary production of 3.3% per decade from 1982 to 1999 (Nemani, 2003) and predicted increases in abundances (and associated change in distribution) of 2.9% per decade for *Prochlorococcus* and 1.4% per decade for *Synechococcus* (Flombaum et al., 2013). They are also of similar scale to the difference between equations proposed to account for the colimitation of phytoplankton growth by temperature and nutrients (Thomas et al., 2017). Differences between equations are smaller than the estimated decline in phytoplankton biomass globally of 10% per decade over the last century (Boyce, Lewis, & Worm, 2010). However, these trends have been disputed (McQuatters‐Gollop et al., 2011), and growth rate and standing phytoplankton biomass are not expected to be correlated (Behrenfeld, 2014).

### 4.2 Constraints on cardinal temperatures

The relatively small difference between equations in the estimates of *T*_opt_ for a given dataset may in part be attributed to the fact that the fitting of these equations has a bias to solutions that return values for the optimal temperature that fall within the temperature range measured or even at the mean temperature. In published datasets (Thomas et al., 2012), the estimates of optimal temperature were correlated with the mean temperature of measurements (*R* ^2 ^= 0.8, Fig. S6). This may reflect a bias of the underlying equation to force *T*_opt_ to approach the mean of the temperatures at which measurements were made. Alternatively, experimentalists may use prior knowledge of temperatures where their species can grow to select experimental temperatures that are centered around *T*_opt_. Finally, negative growth rates may not be reported. However, when fitting all equations to randomly generated data, strong correlations between the midpoint (or mean) of the range in measurement temperatures and calculated *T*_opt_ remain for most equations (Fig. S7). The fact that equations can bias *T*_opt_ toward mean values of the dataset can have important implications for the studies attempting to find a mechanistic explanation for differences in optimal temperatures [e.g., (Sal, Alonso‐Saez, Bueno, Garcıa, & Lopez‐Urrutia, 2015)].

The constraints on *T*_opt_ estimates from equation fits pose a major challenge for the estimation of confidence around estimates of *T*_opt_. Bootstrapping methods (modeling on samples arising from random sampling from the original complete dataset with replacement resulting in equation fits on even smaller subsets of data) commonly used to estimate will greatly underestimate parameter variance. In contrast, Monte Carlo simulations can greatly overestimate the size of the confidence interval around fits because the parameters do not follow an established multivariate distribution that can easily be simulated from the variance/covariance matrices and thus impossibly large or small rates can be predicted from simulations that ignore this issue.

The other cardinal temperatures (CT_min_, CT_max_, *T*_50 min_, *T*_50max_) are less constrained by the temperatures at which measurements were obtained (S7). To ensure accurate estimates of the extreme cardinal temperatures (CT_min_, CT_max_), extremely low growth rates (μ/μ_max_ < 0.05) must be included within the data. This is because the lower and upper thermal tolerance limits (i.e., CTmin and *T*_max_) are less constrained by the mean experimental temperatures than *T*_opt_ and are more dependent on the “shape” implicit in the equations (e.g., sine vs. Gaussian). These limitations may combine to yield a large difference between equations in the estimation of these cardinal temperatures. This may partially explain why correlations between maximal (and minimal temperatures) and ambient temperature or latitude are often absent or weaker than those found for *T*_opt_ in meta‐analyses based on reported cardinal temperatures (Araújo et al., 2013; Sunday et al., 2014), although correlation with latitude of equal strength has been found for *T*_opt_, *T*_min,_ and *T*_max_ when the same equation is applied across all datasets (Thomas et al., 2016).

The larger differences between equations at the upper and lower temperature regions of the curves (*T*_min_, *T*_50 min_, *T*_50max_, *T*_max_) are particularly problematic for the prediction of the response of organisms to global change. In addition to implications of shifts in range limits, these values will influence how an organism can cope with fluctuating temperatures. Increased temperature variation, and thus the capacity to deal with these more extreme temperatures, is expected to pose a greater threat to species survival than warming (Vasseur et al., 2014). Thermal variability can also alter the shape and the scale of the thermal response of organisms (Paaijmans et al., 2013). In a variable environment, based on Jensen's inequality, the optimal mean temperature is expected to be lower than in a constant environment [reviewed in Dowd et al., 2015)] leading to observations of optimal temperature higher than the mean temperature of the environment in more variable temperate habitats compared to less variable tropical habitats (Amarasekare & Johnson, 2017). The temperature response is also dependent on prior exposure to the measurement temperature, allowing for acclimation, and the duration of the exposure (Schulte, Healy, & Fangue, 2011). As a result, temperature fluctuations and acclimation need to be accounted for both in strategies for measurement and potentially in the design of equations.

### 4.3 Implications for evolution under global change

In addition to the difference in estimates of cardinal temperatures, the shape of the temperature response curve will influence many predicted responses (Dowd et al., 2015), including the probability of an evolutionary response to global warming. If the absolute value of the first derivative of the curve (Figure 3) is high (i.e., a steep temperature response, high‐temperature sensitivity, a high Q_10_), a small change in temperature would be expected to lead to a large change in biological process, which in turn would be expected to translate into a large change in selection. The evolutionary outcome of this selection pressure will depend on numerous factors including the standing genetic diversity of the population, the population size, the temperature history of the population (Bell, 2013; Bell & Collins, 2008), and the direction of the change (Low‐Décarie et al., 2014). For example, Equation 15 will predict a steeper temperature response at extreme temperatures than Equation 9 and thus lead to a prediction of greater thermal sensitivity and a higher selection pressure. In fluctuating environments, evolution should lead to a reduction in temperature sensitivity (i.e., an increase in plasticity and a flattening of the response curve; (Clarke & Fraser, 2004).

Across our datasets, activation has a lower slope than inactivation (negative skew). The skew is found to correlate positively with optimal temperature (Pawar et al., 2016), consistent with a fixed upper limit to biological activity. This leads to the expectation of higher selection at the upper limits of thermal tolerance. This is compatible with the observation that the upper limits of heat tolerance in terrestrial ectotherms are highly conserved across taxonomic groups, whereas there is large variation in cold tolerance (Araújo et al., 2013) and that upper limits of heat tolerance correlate with latitude, whereas lower temperature tolerance may not (Sunday et al., 2014).

### 4.4 There is no “Best” equation

Despite the importance of these differences between equations, the best equation for the response to temperature of phytoplankton growth rate or other biological traits cannot be reliably established on a single criterion. Notwithstanding penalties in the metric of equation quality, many equations with higher numbers of parameters had lower BIC, but more complex equations were less robust to loss in data quality. Equation 15 for the response of phytoplankton growth to temperature performed well in terms of fit and robustness to data resolution but not robustness to limitations in the range of relative growth rates captured within the experiment. The fact that we could not identify the “best” equation may be related to important biological phenomena, such as fundamental differences in the shape of the biological response among taxa or among the biological processes of interest, or issues with the data, fitting, and model selection.

The better performance in terms of likelihood of more complex equations suggests that most responses exhibit taxon‐specific patterns, such as skew and concave or convex activation, that must each be captured by a parameter. It may not be possible to have a single best equation. The mechanism of response to temperature of different major taxonomic groups may differ and even the response of different developmental stages for a given taxonomic group may exhibit differences in the shape of their response to temperature (Mordecai et al., 2013; Paaijmans et al., 2013; Sinclair et al., 2016). Even genotypes within a species may differ in the shape of their temperature response (Boyd et al., 2013). For example, the temperature response may fundamentally differ between major groups of phytoplankton (Chen & Laws, 2016; Lürling, Eshetu, Faassen, Kosten, & Huszar, 2013; Thomas et al., 2016). Each major taxonomic group would require an equation that captures these differences in response. Testing this hypothesis would require the measurement of the response to temperature of many minor taxonomic groups (e.g., species) within major taxonomic groups with equally high‐temperature resolution and range coverage for each tested taxon. Alternatively, an equation may yet be developed that outperforms all the equations we have tested, independent of taxa, at least for a given trait. This equation may be based on a better integration of interactions between multiple mechanisms for activation (e.g., accounting for different activation rates of multiple enzymes) and inactivation (heat capacity, substrate availability, and ecological factors) or include a yet to be established mechanistic explanation for these processes.

The limitations of current temperature response data for equation selection have been extensively recognized (Knies & Kingsolver, 2010; Pawar et al., 2016). Our results show that even for a single selected equation, very few existing datasets meet data quality requirements to minimize error in predictions of cardinal temperatures and rates across the full biokinetic temperature range. For recovering estimates from existing data that are limited by resolution and range, a robust equation with few parameters (e.g., Equation 8) that may not accurately represent the underlying process and patterns (such as skew) is preferable to better fitting equations for which changes in data range and resolution lead to important changes in estimates (e.g., Equation 6). We did not vary the precision of the measurement of rate or of temperature. A proposed rule of thumb is that the precision of the measurement of temperature is at least three times that of the precision of the measurement of the response variable (Pawar et al., 2016). Another element not tested in our analysis is the location along the temperature scale, although measured activation can differ between organisms with colder or warmer growth ranges (Pawar et al., 2016), potentially influencing model choice, but this could not be tested in our high‐resolution datasets because of temperature ranges for growth mostly overlapped. The challenge of model selection and the lack of quality data limit our ability to predict, for example, changes in the distribution of species with global climate change [e.g., (Gobler et al., 2017)].

Even in simple laboratory experiments with only a single trophic level, the response to temperature of growth rate does not consistently lead to predictable changes in competitive dynamics (Limberger, Low‐Décarie, & Fussmann, 2014). While the biogeography of marine ectotherms matches the predictions of their thermal performance curves, this is not the case for terrestrial ectotherms (Sunday, Bates, & Dulvy, 2012). These differences between the response of species to temperature, competition, and their distribution may be attributed to the complexities of ecological interactions and the associated need to integrate many concomitant biological responses with the potential for nonlinear interactions. These differences may also limit the credibility of biogeographic inferences such as that presented in Figure 6, which would completely change if, for example, nutrient limitation was included (Thomas et al., 2017). In models of natural ecosystems, the difference in response between trophic levels can cause trophic cascades, exacerbating the predicted effect of warming (Chust et al., 2014). However, these differences between single species physiological responses and ecological observations may in part be resolved by a better measurement and understanding of the individual species responses to temperature. Our findings highlight the need to focus our measurement and modeling efforts on simple but fundamental aspects of the response of organisms to temperature, with the aim to make more robust predictions on the changes in the ecology of organisms and associated global biogeochemical processes based on future climate scenarios.

## DATA AVAILABILITY STATEMENT

Data and scripts are available on Dryad (https://doi.org/10.5061/dryad.52mc5).

## CONFLICT OF INTEREST

None declared.

## AUTHOR CONTRIBUTION

Tobias Boatman, Noah Bennett, Will Passfield, Antonio Gavalás‐Olea, and Philipp Siegel acquired the experimented data. Noah Bennett and Etienne Low‐Décarie implemented the coding. Richard Geider and Etienne Low‐Décarie designed the research and wrote the manuscript. Etienne Low‐Décarie, Tobias G. Boatman, Noah Bennett, Will Passfield, Antonio Gavalás Olea, Philipp Siegel, and Richard J. Geider edited and critically reviewed the manuscript.
