# Supplementary material for: Predictions of response to temperature are contingent on model choice and data quality
Source: Ecol Evol. 2017 Nov 15;7(23):10467–81. doi: 10.1002/ece3.3576 (PMC5723626; doi:10.1002/ece3.3576)
Supplement: Supplementary file 1 [file ECE3-7-10467-s001.docx]

**Supporting Information**

**Table of content**

Equations for sub-optimal temperatures 2

Mean growth as a function of temperature in seven phytoplankton genera (Fig. S1) 4

AIC and AICc results (FIG. S2) 5

Model clustering based on similarity of prediction (FIG. S3) 6

Simulation: fitting models to data simulated using the models (FIG. S4) 7

Model deviations from weighted mean (FIG. S5) 8

Correlation between Topt and mean temperature: published data (FIG. S6) 9

Correlation between Cardinal Temperatures and Measurement Temperature: simulation (FIG. S7) 10

References for supporting information 11

# Equations for sub-optimal temperatures

The response to suboptimal temperatures is usually assumed to be driven by the thermodynamics of chemical reactions. The Arrhenius equation was originally derived to describe the temperature dependence of biochemical reactions, but has also been applied to physiological rates (Equ. 1). In Equ. 1, k(T) is the reaction rate constant, *a* is a pre-exponential term that serves as a scaling factor, R is the universal gas constant, E_A_ is the activation energy of the reaction, and T is the absolute temperature in K. This equation is sometimes presented in this alternative form (Equ. 2), which is convenient when comparing rates at two temperatures to obtain a Q_10_. In Equ. 2, k(T_ref_) is the rate at the reference temperature (T_ref_), A is parameter that accounts for the T-dependence (note A = E_A_/R). Another equation that has also been used to account for the increase of rate with temperature in the region between T_min_ and T_opt_ is an exponential function that is analogous to the van Hoft equation (Equ. 3); where T is the temperature (°C), k_0_ is the rate at 0 °C and k is a fitted parameter.

The quality of fit of the Arrhenius equation (Equ, 1 or 2) can be affected by the range of data selected, which requires that caution be used when using numerical values of the activation energy to support or refute the thermodynamic effect of increases of temperature on reaction rates (Knies & Kingsolver, 2010; Pawar *et al.*, 2016). For example, it is often observed that a decrease in temperature sensitivity (i.e., Q_10_ or first derivative) as temperatures approach T_opt_. This may be a consequence of T-dependent changes in substrate availability in Michaelis–Menten kinetics. Specifically, if the substrate concentration required for maximal rates and the maximal rate itself are both positively correlated with temperatures, substrate limitation will increase with temperature. For example, temperature dependence of decomposition in soil may be contingent on substrate availability (Davidson *et al.*, 2006) and nutrient concentration can alter the optimal temperature for phytoplankton growth (Thomas *et al.*, 2017). Other processes than substrate availability may need to be included to accurately modeled the inactivation processes at supra-optimal temperatures. Another conceptual limitation of the thermodynamic interpretation is that adaptation to temperature conditions across species or genotypes can lead to isoenzymes with equal rates at their adaptive temperature (Clarke & Fraser, 2004) (in a species adapted to a lower temperature, a given enzyme can reach the same rates at this lower temperature than the homologous enzyme in an organism adapted to a higher temperature found at that higher temperature). By harnessing these different temperature adapted forms, an invariant rate with temperature should be possible, at least across a given range.

Table 1: Equation for sub-optimal temperatures

| Formula | Equ. | Number of Parameters | Reference |
| --- | --- | --- | --- |
| $Rate= a\cdot exp\left( \frac{-E_{A}}{R\cdot T} \right)$ | 1* | 2 | (Raven & Geider, 1988) |
| $Rate=k(T_{ref})\cdot exp\left\lceil-\frac{E_{A}}{R}\left( \frac{1}{T}-\frac{1}{T_{ref}} \right) \right\rceil$ | 2* | 2 | e.g. (Li *et al.*, 1984; Geider *et al.*, 1997) |
| $Rate= k_{0}\cdot exp\left( k\cdot T \right)$ | 3* | 2 | (Eppley, 1972) |

# Mean growth as a function of temperature in seven phytoplankton genera (Fig. S1)

Fig. S1: Mean growth as a function of temperature in seven phytoplankton genera.

# AIC and AICc results (FIG. S2)

Figure S2 Rank of each model based on AIC and AICc value. Models are order by median rank. Point is the median and error bars are 95% confidence interval across datasets.

# Model clustering based on similarity of prediction (FIG. S3)

Figure S3 Relationship between all models based on distance between predicted values at each temperature across all phytoplankton growth datasets. Number of parameters are in parentheses.

# Simulation: fitting models to data simulated using the models (FIG. S4)

To ensure that the high-resolution data sets were of sufficient quality to distinguish between equations, we conducted a simulation based on equation fits to each dataset. Normally distributed random noise was added to the predicted growth rate value from each equation at each temperature. The noise was centered on 0 and its standard deviation was the square root of the mean residuals squared arising from the fit of the equation. Each equation was then fit to the simulated datasets generated by each equation and ranked based on BIC. Each simulation was replicated 5 times.

a) b)

Fig. S4 Width of vertical ribbons indicate density across all datasets and replications. a) Rank of models used to generate the data (eg. rank of model 4 on data generated using model 4). Density is largest near 1 except for model 5. b) Median rank of models not used to generate the data (eg. median rank of model 5-15 on data generated using model 4).

Simulations indicate that the quality of the phytoplankton growth datasets is sufficient to differentiate between equations. All equations had better rankings on the simulated data that they had generated than on data generated by any other equation (Kruskal-Wallis p< 10^-3^).

# Model deviations from weighted mean (FIG. S5)


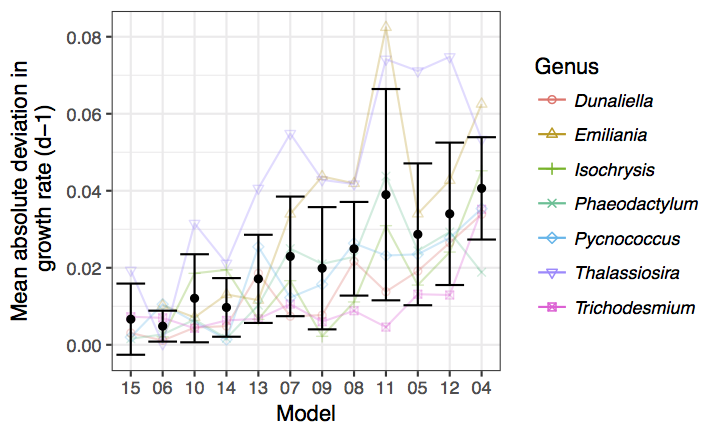
a) b)

Figure S5 For each model, **a)** the deviation of T_opt_ predicted by the model from the weighted mean T_opt_ (°C) across all models (weights are Akaike weights). Only data that was within the 95% confidence interval of the median across models is presented**. b)** Mean absolute deviation in the predicted growth rate at each temperature compared to the weighted mean across models (weights are Akaike weights).

# Correlation between Topt and mean temperature: published data (FIG. S6)

Fig. S6: Relationship between calculated optimal temperature for phytoplankton growth and the mean experimental temperature for 130 species of phytoplankton (Thomas et al., 2012). For the growth response of phytoplankton to temperature 80% of the variance in the T_opt_ can be accounted for by the experimental temperature values alone, i.e. without any rate measurement (blue continuous line is the linear regression, the dashed line is the 1:1 line).

# Correlation between Cardinal Temperatures and Measurement Temperature: simulation (FIG. S7)

a) b)

Fig. S7 a)s Comparison of modelled T_opt_ and mean temperature of the simulated dataset to which the model was applied. Simulated experiments with a range of 10°C measured at each 0.1°C and a mean value between 0 and 30°C were generated with growth rate generated by a uniform random distribution between 0 and 1 (values of 0 are removed before fitting the equations).

b) Comparison of modelled T_50min_ and minimum temperature of the simulated dataset to which the model was applied (similar lack of correlation arises for other non-central parameters and data summary statistics). Simulated experiments with a range of 10°C measured at each 0.1°C and a mean value between 0 and 30°C were generated with growth rate generated by a uniform random distribution between 0 and 1 (values of 0 are removed before fitting the equations).

# References for supporting information

Clarke A, Fraser KPP (2004) Why does metabolism scale with temperature? *Functional Ecology*, **18**, 243–251.

Davidson EA, Janssens IA, Marks D et al. (2006) Temperature sensitivity of soil carbon decomposition and feedbacks to climate change. *Nature*, **440**, 165–73.

Eppley RW (1972) Temperature And Phytoplankton Growth In The Sea. *FISHERY BULLETINF.*, **70**, 1063–1085.

Geider RJ, MacIntyre HL, Kana TM (1997) Dynamic model of phytoplankton growth and acclimation: Responses of the balanced growth rate and the chlorophyll a:carbon ratio to light, nutrient-limitation and temperature. *Marine Ecology Progress Series*, **148**, 187–200.

Knies JL, Kingsolver JG (2010) Erroneous Arrhenius: modified Arrhenius model best explains temperature dependence of ectotherm fittness. *The American Naturalist*, **176**, 227–233.

Li W, Smith J, Piatt T (1984) Temperature response of photosynthetic capacity and carboxylase activity in Arctic marine phytoplankton. *Marine Ecology Progress Series*, **17**, 237–243.

Pawar S, Dell AI, Savage VM, Knies JL (2016) Real versus Artificial Variation in the Thermal Sensitivity of Biological Traits. *The American Naturalist*, **187**, E41–E52.

Raven JA, Geider RJ (1988) Temperature and algal growth. *New phytologist*, **110**, 441–461.

Thomas MK, Kremer CT, Klausmeier C a., Litchman E (2012) A Global Pattern of Thermal Adaptation in Marine Phytoplankton. *Science*, **338**, 1085–1088.

Thomas MK, Aranguren-Gassis M, Kremer CT, Gould MR, Anderson K, Klausmeier CA, Litchman E (2017) Temperature-nutrient interactions exacerbate sensitivity to warming in phytoplankton. *Global Change Biology*, **3**, 1375–1387.
